# Laser peripheral iridectomy use in the unoperated eyes of hospitalised patients with primary angle-closure glaucoma in a national multicentre study in China

**DOI:** 10.1186/s12886-026-04873-y

**Published:** 2026-05-07

**Authors:** Huiyan Mao, Kun Xiong, Yongzhen Yu, Qi’ao Zhang, Jinyuan Chen, Xue Yin, Dan Wang, Hong Sun, Xiaoli Xing, Guoping Duan, Zhiyang Jia, Jian Jiang, Zhengzheng Wu, Li Tang, Peng Lu, Danyan Liu, Yajuan Zheng, Lidong Zhuo, Sujie Fan, Xinying Zhang, Weiwei Liu, Yan Dai, Hong Chen, Huadong Xiang, Jingyi Lv, Yang Yang, Jianjun Ma, Jianfang Yang, Xueli Cao, Tingting Zhou, Wenyi Guo, Guoxing Li, Shaodan Zhang, Xin Sun, Mingguang He, Yuanbo Liang

**Affiliations:** 1 National Clinical Research Center for Ocular Diseases, Eye Hospital, Wenzhou Medical University, Wenzhou, Zhejiang 325027 China; 2 Aier Eye Hospital of Wuhan University, Wuhan, 430061 China; 3 The Affiliated Eye Hospital, Jiangxi Medical College, Nanchang University, Jiangxi Clinical Research Center for Ophthalmic Disease, Nanchang, 330006 China; 4 Department of Ophthalmology, The First Affiliated Hospital, Fujian Medical University, Fuzhou, 350000 China; 5 First Affiliated Hospital of Soochow University, Suzhou, Jiangsu 215000 China; 6 Jilin Province People’s Hospital, Changchun, Jilin 130000 China; 7 Jiangsu Province People’s Hospital, Nanjing, Jiangsu 210000 China; 8 Glaucoma, Tianjin Medical University Eye Hospital, Tianjin, 300000 China; 9 Hunan Provincial People’s Hospital, Changsha, Hunan 410000 China; 10 Hebei General Hospital, Shijiazhuang, Hebei 050000 China; 11 Eye Center, Xiangya Hospital of Central South University, Changsha, Hunan 410000 China; 12 Sichuan Provincial People’s Hospital, Chengdu, Sichuan 610000 China; 13 Department of Ophthalmology, West China Hospital of Sichuan University, Chengdu, Sichuan 610000 China; 14 Lanzhou University Second Hospital, Lanzhou, Gansu 730000 China; 15 The Second Hospital of Hebei Medical University, Shijiazhuang, Hebei, Shijiazhuang 050000 China; 16 Jilin University Second Hospital, Changchun, Jilin 130000 China; 17 Liaoyuan City Central Hospital, Liaoyuan, Jilin 136000 China; 18 Department of Glaucoma, Handan City Eye Hospital, Handan, Hebei 056000 China; 19 Jilin Central General Hospital, Changchun, Jilin 130000 China; 20 Huaiyin Hospital of Huai’an City, Huai’an, Jiangsu 223001 China; 21 Mianyang Central Hospital, Mianyang, Sichuan 621000 China; 22 Hebei Eye Hospital, Xingtai, Hebei 054000 China; 23 Zhangjiajie People’s Hospital, Zhangjiajie, Hunan 427000 China; 24 Lixiang Eye Hospital of Soochow University, Suzhou, Jiangsu 215000 China; 25 Yueyang Central Hospital, Yueyang, Hunan 414000 China; 26 Department of Ophthalmology, Gansu Provincial Hospital, Lanzhou, Gansu 730000 China; 27 Ziyang Hospital of Traditional Chinese Medicine, Ziyang, Sichuan 641300 China; 28 Baiyin City Central Hospital, Baiyin, Gansu 730900 China; 29 Zhangye People’s Hospital, Zhangye, Gansu 734000 China; 30 Ophthalmology, Shanghai Ninth People’s Hospital, Shanghai Jiaotong University School of Medicine, Shanghai, 200000 China; 31 Sichuan University West China Hospital, Chengdu, Sichuan 610000 China; 32 State Key Laboratory of Ophthalmology, Zhongshan Ophthalmic Center, Sun Yat-sen University, Guangzhou, 510060 China; 33 Experimental Ophthalmology, The Hong Kong Polytechnic University, Hong Kong, 999077 People’s Republic of China; 34 Department of Glaucoma, National Clinical Research Center for Ocular Diseases, Eye Hospital, Wenzhou Medical University, No. 270, Xue Yuan West Road, Wenzhou, 325027 China

**Keywords:** Primary angle-closure glaucoma, Laser peripheral iridectomy, Multi-centre study

## Abstract

**Objective:**

To investigate the use of laser peripheral iridectomy (LPI) in the unoperated eyes of hospitalised patients with primary angle-closure glaucoma (PACG).

**Methods:**

This was a national multi-centre retrospective study, and the sample was selected via multi-stage stratified random sampling from 26 hospitals. Data on LPI use were extracted from the electronic medical records (EMRs) of 5,404 PACG patients from 1 January 2011 to 31 December 2020. We used a generalised estimating equations (GEE) model to identify factors influencing LPI use.

**Results:**

Among 3,525 unoperated eyes of hospitalised PACG patients, 35.3% of these eyes received LPI during hospitalisation, and the usage rate was 35.1% and 36.0% in provincial-level or above and in city-level hospitals, respectively. In multivariable models, an intraocular pressure (IOP) of > 21 mmHg (OR = 0.915; 95% CI [0.875, 0.956]), moderate visual impairment (OR = 0.898; 95% CI [0.849, 0.949]) and severe visual impairment or blindness (OR = 0.755; 95% CI [0.717, 0.794]) were associated with a lower LPI usage rate in unoperated eyes. By contrast, patients treated in the northern region had a higher LPI usage rate in unoperated eyes (OR = 1.343; 95% CI [1.295, 1.393]).

**Conclusions:**

The usage rate of LPI in the unoperated eyes of PACG patients is relatively low in China, especially among patients in the southern region, with higher IOP and worse visual impairment.

**Supplementary Information:**

The online version contains supplementary material available at 10.1186/s12886-026-04873-y.

## Introduction

Glaucoma is one of the leading cause of irreversible blindness worldwide. In 2020, there were essentially 76 million people with glaucoma globally, and it is expected to increase to 112 million by 2040 [1]. Primary angle-closure glaucoma (PACG) is a major subtype of glaucoma, and China has the largest population of PACG patients worldwide, with an estimated 10 million cases, accounting for approximately three-quarters of the global total [2]. Moreover, the blindness rate of PACG is high, about three times that of primary open-angle glaucoma [3]. Timely intervention for PACG is crucial to prevent its progression and irreversible blindness.

Since the middle of the 1970s, laser peripheral iridotomy (LPI) has been used in clinics and endorsed as the first treatment strategy for PACG by multiple clinical guidelines [4–6]. LPI can relieve pupillary block, allowing the convex iris to flatten and broaden the anterior chamber angle [7]. Long-term clinical trials have validated its efficacy: The Zhongshan Angle-Closure Prevention study showed that LPI achieved a nearly 50% reduction in the risk of progression from primary angle-closure suspect to primary angle-closure in six years [8]. Additionally, LPI has been proven to effectively reduce anti-glaucoma medication and surgical needs. In one study, 77.1% of patients required no medications post-LPI and had low rates of cataract (10.1%) or glaucoma surgery (4.8%) within 2 years after LPI [9]. These confirmed benefits make LPI critical for the unoperated eyes of hospitalised PACG patients.

Notably, the fellow eyes of patients with acute PACG carry a high risk (50%–80%) of an acute attack within 5 years [10]. Even with long-term miotic therapy, the probability of an acute attack in these eyes remains at 40% within 5 years [11]. Since a subset of hospitalised PACG patients presents with acute PACG, their unoperated eyes (often the fellow eyes of the attacked eyes) are exposed to this high risk. Therefore, we believe that the unoperated eyes of hospitalised PACG patients should undergo LPI.

Despite this evidence and clear guideline recommendations, the actual use of LPI in the unoperated eyes of hospitalised PACG patients remains unclear. Previous studies on PACG prophylaxis have focused on high-risk populations screened in the community rather than on hospitalised PACG patients [12, 13]. Nevertheless, hospitalised PACG patients may present with clearer clinical signs and treatment indications than community populations. Performing LPI in these unoperated eyes can more directly reflect the discrepancy between guidelines and real-world clinical practice. The China Glaucoma Treatment Pattern Study Ⅰ–Primary Angle-Closure Glaucoma (Ch-GTPⅠ) [14, 15] is a national multi-centre retrospective study of hospitalised patients with PACG. This study aimed to analyse the use status of LPI in the unoperated eyes of hospitalised PACG patients in China.

## Methods

### Study design and patients

The protocol of this national multicentre retrospective study has been described in detail in previous studies [14, 15]. To ensure that a nationally representative sample would be obtained, a multi-stage stratified sampling method was used to select the participants. Briefly, one province was randomly selected in each region (North, Northeast, East, Central, Southwest, and Northwest). A university-affiliated hospital and a provincial people’s hospital were selected from each province. Two cities and four counties were randomly selected from each province. A central hospital or a specialised eye hospital in the selected cities, as well as county hospitals in each county were included. Additionally, we randomly selected two hospitals from among the top 10 ophthalmic centres in China. We then used systematic random sampling methods to choose patients with PACG from the electronic medical record (EMR) system at each chosen hospital from 1 January 2011 to 31 December 2020. The number of cases to be selected per year was allocated proportionately based on the hospital caseload. This study protocol was approved by the Eye Hospital of Wenzhou Medical University’s ethics committee (2021–126 K-108-01). Given that the study was conducted retrospectively, written informed consent from the patients was not required by the ethics committee (Eye Hospital of Wenzhou Medical University’s ethics committee).

The sample size was calculated as described in the previously published protocol [14]. Three hundred cases were enrolled at the top 10 ophthalmic hospitals and each provincial-level hospital, as well as 150 cases from each city-level hospital and 75 cases from each county-level hospital. However, we failed to identify sufficient cases from county-level hospitals due to the small number of patients presenting with PACG and the incompleteness of their EMR systems. Ultimately, 26 hospitals participated. The number of cases enrolled in each hospital is shown in Table S1.

### Definition of primary angle-closure glaucoma and study population

PACG was diagnosed based on the 10th Revision of the International Classification of Diseases codes and diagnosis terms (PACG, acute PACG and chronic PACG) in the medical record. In this study, “hospitalised patients” specifically refers to individuals who were admitted for inpatient care due to a primary diagnosis of PACG, where the primary purpose was to treat glaucoma during the study period (1 January 2011 to 31 December 2020).All patients 18 years or older diagnosed with PACG were included in this study. The exclusion criteria were (1) prior glaucoma surgery or LPI; (2) other previous intraocular surgeries, such as operations for ocular trauma and vitreoretinal surgery; (3) other ocular disease contraindications to LPI, such as corneal leukoplakia, iridocorneal endothelial syndrome, corneal endothelial decompensation and uveitis. The “unoperated eyes” refers to eyes diagnosed with PACG but without any glaucoma operation, cataract operation, or combined glaucoma and cataract operation prior to or during hospitalisation. A history of previous LPI was also considered as an operation.

### Definitions of laser peripheral iridectomy (LPI), vision impairment and peripheral anterior synechiae

We determined whether a patient had received LPI before or during hospitalisation based on whether the LPI or LPI plus laser peripheral iridoplasty (LPIP) procedure was recorded in their EMR. We classified vision impairment based on logMAR visual acuity as follows [16]: no vision impairment (logMAR visual acuity ≤ 0.3), mild vision impairment (logMAR visual acuity ≤ 0.5 and > 0.3), moderate vision impairment (logMAR visual acuity ≤ 1.0 and > 0.5), severe vision impairment or blindness (logMAR visual acuity > 1.0). peripheral anterior synechiae (PAS) was defined as the adhesion of the iris to the trabecular meshwork wider than half a clock-hour according to the gonioscopy [17]. Patients with different extents of PAS were divided into two groups – group A: PAS ≤ 180 degrees (≤ 6 clock-hours) and group B: PAS > 180 degrees (> 6 clock-hours).

### Data collection

Trained interviewers extracted data of patients’ demographic and clinical characteristics from EMR. Demographic data included age, gender (male/female) and residence (urban/rural). Clinical characteristics included intraocular pressure (IOP), number of anti-glaucoma drugs, axial length, visual acuity, history of diabetes, hypertension, previous glaucoma and ocular surgery.

### Statistical analysis

Continuous variables with a normal distribution are expressed as means and standard deviations, and variables not conforming to a normal distribution are expressed as medians and interquartile ranges. Categorical variables are expressed as numbers and percentages. Continuous variables were compared using an independent -samples t-test or Mann -Whitney U test, and categorical variables were compared using a chi-square test. The generalized estimating equations (GEE) model was adopted to determine the factors associated with LPI use. Variables with statistical significance (*p* < .05) in the univariate analysis were further included in the multivariate analysis. All statistical analyses were performed using Stata version 17.0 (Stata Corporation, College Station, Texas, USA). Statistical significance was defined as a *p-value* of < 0.05.

## Results

### Baseline characteristics of patients who received LPI in unoperated eyes

**Fig. 1:**
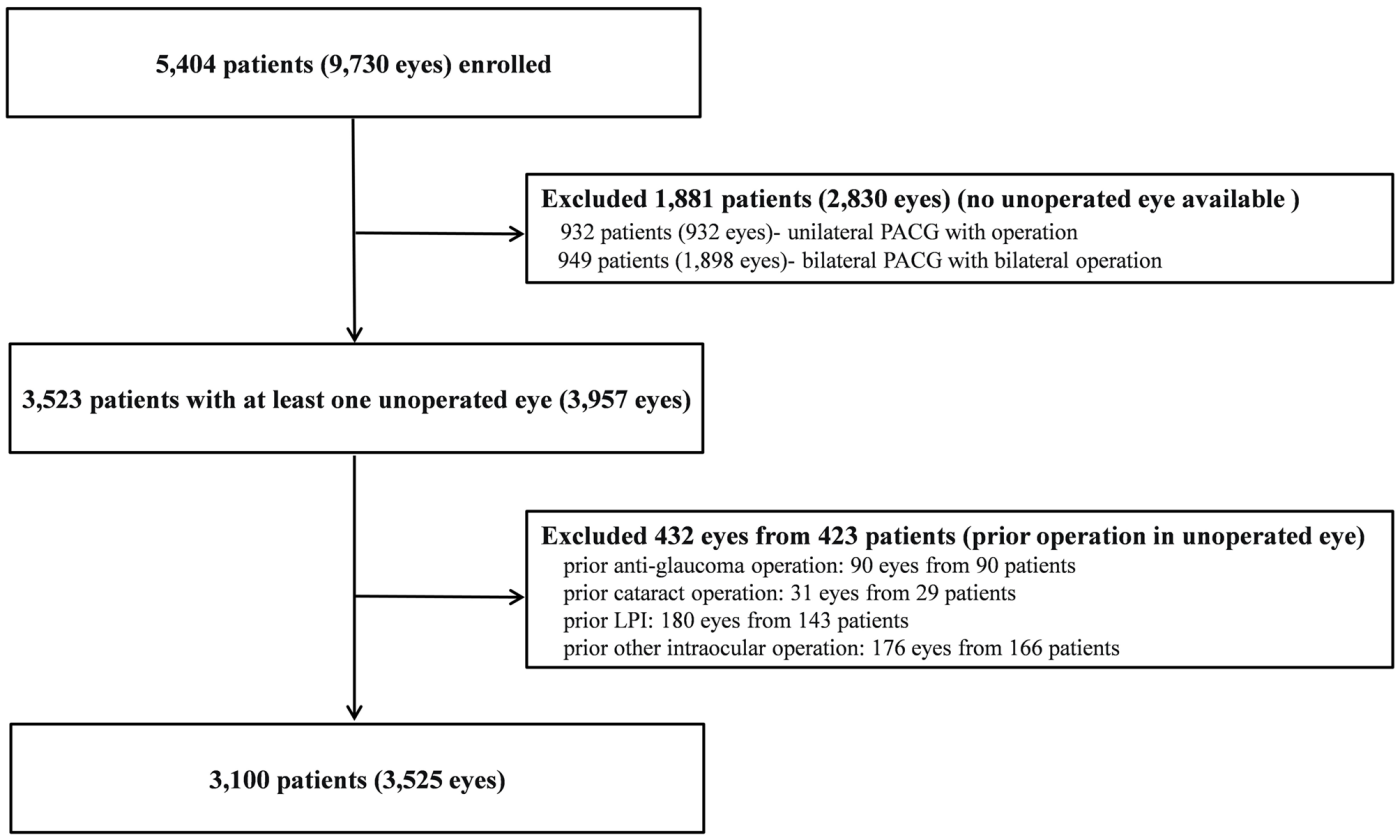
Flow chart

Among all PACG eyes, 5,864 eyes had received definitive surgical interventions during hospitalisation. According to clinical guidelines and routine ophthalmic practice in China [18], such operated cases typically present with advanced disease, uncontrolled IOP, extensive PAS, or complicated concurrent cataracts. As definitive surgical treatment had already been administered, these eyes were excluded from the subsequent LPI-related analysis. A total of 3,100 patients (3,525 eyes) were enrolled (Fig. 1), of whom 1,081 (34.9%) patients, with 1,246 (35.3%) eyes, received LPI. Patients who did not receive LPI were older than those who received LPI (65.7 vs. 64.3 years, *p* <.001). Generally, compared to patients who received LPI, those who did not receive LPI were less likely to have hypertension or cardiovascular disease and had a higher proportion of rural residents (*p* <.05). The latter group also had a higher IOP (20.6 mmHg vs. 18.1 mmHg, *p* <.001), worse visual acuity, and fewer number of anti-glaucoma drugs (Table 1).

**Table 1:** Demographic and clinical characteristics of the primary angle-closure glaucoma patients

	Total sample	LPI		*P*-value
	*n* = 3,100	Yes (*n* = 1,081)	No (*n* = 2,019)	
Age	65.2 ± 9.6	64.3 ± 8.9	65.7 ± 9.9	< 0.001
Gender (Male)	964 (31.1)	316 (29.2)	648 (32.1)	0.101
Type of Hospital (provincial-level or above)	2,229 (71.9)	765 (70.8)	1,464 (72.5)	0.303
Married (yes)	2,764 (95.5)	1,006 (96.0)	1,758 (95.3)	0.375
Systemic diseases				
Hypertension (yes)	1,026 (33.1)	418 (38.7)	608 (30.1)	< 0.001
Diabetes(yes)	276 (8.9)	100 (9.3)	176 (8.7)	0.619
Cardiovascular (yes)	276 (8.9)	112 (10.4)	164 (8.1)	0.037
Age				
< 45	72 (2.5)	19 (1.9)	53 (2.8)	< 0.001
45–54	353 (12.2)	135 (13.4)	218 (11.5)	
55–64	954 (32.9)	363 (36.1)	591 (31.1)	
65–74	1,001 (34.5)	347 (34.5)	654 (34.4)	
75+	524 (18.0)	141 (14.0)	383 (20.2)	
Residence (urban)	1,346 (49.2)	541 (52.7)	805 (47.1)	0.005
Intraocular pressure (mmHg)	19.7 ± 11.4	18.1 ± 9.3	20.6 ± 12.3	< 0.001
LogMAR visual acuity				
No vision impairment	1,713 (56.2)	702 (65.5)	1,011 (51.1)	< 0.001
Mild vision impairment	480 (15.7)	178 (16.6)	302 (15.3)	
Moderate vision impairment	430 (14.1)	146 (13.6)	284 (14.4)	
Severe vision impairment or blindness	426 (14.0)	46 (4.3)	380 (19.2)	
Number of anti-glaucoma drugs				
0	1,695 (54.7)	601 (55.6)	1,094 (54.2)	< 0.001
1	423 (13.7)	193 (17.9)	230 (11.4)	
2	387 (12.5)	150 (13.9)	237 (11.7)	
3+	595 (19.2)	137 (12.7)	458 (22.7)	

### Performing LPI in unoperated eyes

A total of 1,246 eyes (35.3%) received LPI (Table 2). The results show that the usage rate in provincial-level hospitals or above (35.1%) did not differ significantly from the usage rate in city-level hospitals (36.0%); however, the usage rate of LPI plus LPIP in city-level hospitals (10.4%) was significantly higher than in provincial-level hospitals or above (3.7%). Gonioscopy data were available for 393 eyes from 376 patients. Among these, patients with PAS of ≤ 180 degrees were more likely to receive LPI than those with PAS of > 180 degrees (53.0% vs. 28.2%). When PAS was ≤ 180 degrees, the usage rate of LPI in city-level hospitals (76.1%) was significantly higher than in provincial-level hospitals or above (29.5%). When PAS was > 180 degrees, the usage rate of LPI in city-level hospitals (30.0%) was slightly higher than in provincial-level hospitals or above (26.3%) (Table 3).

**Table 2:** Usage rate of LPI in the unoperated eyes of primary angle-closure glaucoma patients during hospitalisation

	Laser type	Total
All	Total	35.3% (1,246/3,525)
	LPI	29.6% (1,043/3,525)
	LPI plus LPIP	5.8% (203/3,525)
Provincial -level hospital or above	Total	35.1% (863/2,461)
	LPI	31.3% (771/2,461)
	LPI plus LPIP	3.7% (92/2,461)
City-level hospital	Total	36.0% (383/1,064)
	LPI	25.6% (272/1,064)
	LPI plus LPIP	10.4% (111/1,064)

Abbreviations: LPI = laser peripheral iridectomy; LPIP = laser peripheral iridoplasty

**Table 3:** Usage rate of LPI in the unoperated eyes of primary angle-closure glaucoma patients with different extent of PAS

		Total
PAS of ≤ 180 degrees	Total	53.0% (167/315)
	Provincial level- hospital or above	29.5% (46/156)
	City-level hospital	76.1% (121/159)
PAS of > 180 degrees	Total	28.2% (22/78)
	Provincial level- hospital or above	26.3% (10/38)
	City-level hospital	30.0% (12/40)

Abbreviations: LPI = laser peripheral iridectomy; PAS = peripheral anterior synechiae

### Factors associated with LPI use

Table 4 shows univariable and multivariable GEE models for unoperated eyes that received LPI. Gender, age, hospital type, region, residence, IOP, logMAR visual acuity and anti-glaucoma medication were included in the multivariable model. We classify North, Northeast and Northwest as the northern region, and East, Central and Southwest as the southern region. The northern region (OR = 1.343; 95% CI [1.295, 1.393]; *p* < .001) had a higher LPI usage rate in unoperated eyes. By contrast, IOP > 21 mmHg (OR = 0.915; 95% CI [0.875, 0.956; *p* < .001), moderate visual impairment (OR = 0.898; 95% CI [0.849, 0.949]; *p* < .001), and severe visual impairment or blindness (OR = 0.755; 95% CI [0.717, 0.794]; *p* < .001) were associated with a lower LPI usage rate in unoperated eyes.

**Table 4:** GEE analysis of LPI in the unoperated eye of primary angle-closure glaucoma patients

Variables	Univariate regression		Multivariate regression	
	OR (95% CI)	*P*-value	OR (95% CI)	*P*-value
Gender				
Male	Reference			
Female	1.031 (0.994–1.069)	0.098		
Age	0.997 (0.995–0.998)	< 0.001	0.998 (0.996-1.000)	0.064
Residence				
Urban	Reference		Reference	
Rural	0.949 (0.915–0.984)	0.005	0.990 (0.953–1.027)	0.583
Type of hospital				
Provincial-level hospital or above	Reference			
City-level hospital	1.020 (0.982–1.059)	0.306		
Area				
Southern region	Reference		Reference	
Northern region	1.365 (1.413–1.319)	< 0.001	1.343 (1.295–1.393)	< 0.001
Intraocular pressure(mmHg)				
<=21	Reference			
> 21	0.856 (0.826–0.886)	< 0.001	0.915 (0.875–0.956)	< 0.001
Visual acuity				
No vision impairment	Reference		Reference	
Mild vision impairment	0.962 (0.916–1.010)	0.120	0.977 (0.925–1.033)	0.416
Moderate vision impairment	0.932 (0.886–0.980)	0.006	0.898 (0.849–0.949)	< 0.001
Severe vision impairment or blindness	0.739 (0.712–0.768)	< 0.001	0.755 (0.717–0.794)	< 0.001
Number of anti-glaucoma drugs	0.989 (0.973–1.005)	0.160		

Abbreviations: OR = odds ratio; 95% CI = 95% confidence interval

## Discussion

LPI is known to be a vital treatment for PACG, especially in unoperated eyes. This study found that only 35.3% of unoperated eyes received LPI in PACG patients during hospitalisation. Moreover, the use of LPI in the unoperated eyes shows regional variation and is associated with IOP and visual acuity. To the best of our knowledge, this is the first multi-centre study to investigate LPI use in the unoperated eyes of hospitalised PACG patients. Our findings indicate that a considerable proportion of unoperated PACG eyes do not receive timely treatment, which may be a major contributor to the high blindness rate in China [19].

Few studies, especially in China, have reported on the use of LPI in the unoperated eyes of PACG patients. Previous studies have shown that nearly 50,000 LPI procedures are carried out annually in the USA [20], and 10,284 laser iridotomies were performed in the UK in the 2014–2015 period [21]. In Australia, the number of laser iridotomies increased 281%, from 3,785 in 1994 to 14,427 in 2014 [22]. In China, surgical peripheral iridotomy decreased from 9.03% in 2015 to 6.34% in 2019 [23], and the annual rate of LPI due to primary angle-closure disease gradually increased from 8.0/100,000 (*n* = 109) in 2011 to 224.8/100,000 (*n* = 3,704) in 2021 in an urban population [24]. However, national-scale data on LPI use in the unoperated eyes of hospitalised PACG patients remain scarce.

Our results show that the usage rates of LPI in unoperated eyes in provincial-level hospitals or above and city-level hospitals were similar despite the uneven distribution of medical resources in China [25, 26]. This may stem from the relatively low technical threshold for using LPI. This indicates that the implementation of LPI is not restricted by hospital level and that most hospitals are qualified to perform LPI. The usage rates of LPI alone and LPI plus LPIP varied across hospitals. Only 38.1% of Chinese PACG patients had pure pupillary block [27], and more than one-third of PACG patients were diagnosed with plateau iris [28]. These studies support the idea that non-pupillary block mechanisms also play a prominent role in angle closure among Asian populations. In theory, LPI plus LPIP should obtain better outcomes in non-pupillary block patients. Indeed, a prior study confirmed that LPI plus LPIP achieved a greater reduction in PAS extent than LPI alone in PAC/PACG [29]. Regrettably, no high-level evidence currently exists to guide laser selection in various mechanisms of angle closure. A multicentre study by the Multi-mechanism Angle Closure Study group may provide more definitive evidence in the future for laser selection [30].

Our results show that patients with PAS of ≤ 180 degrees were associated with a higher LPI usage rate. While this aligns with early clinical practice patterns in China, LPI is preferred in PACG with PAS of ≤ 180 degrees and trabeculectomy in PAS of > 180 degrees [31]. This may be attributed to the greater effectiveness of LPI in early PACG and its diminished effectiveness in some advanced PACG cases [32]. Our findings suggest that Chinese ophthalmologists tend to regard PAS extent as a key reference for PACG treatment selection, which may reflect a practical consensus on matching intervention strategies to disease severity in clinical practice. However, this retrospective study is limited by incomplete gonioscopy documentation, with only 393 eyes in 376 patients having recorded gonioscopic data, which restricts our ability to understand the mechanisms underlying the making of clinical decision. The identified factors, such as IOP and visual acuity, likely serve as proxies for gonioscopy data rather than independent drivers of treatment decisions. Future studies with systematic gonioscopic assessment are needed to clarify these associations.

We found regional disparities in LPI use, with higher usage rates in the northern region. Interestingly, a survey conducted in 2023 showed that ophthalmologists in the south were more likely to choose LPI for primary angle-closure suspect than those in north [33]. This discrepancy can likely be attributed to differences in the study populations. Regardless, this inconsistency underscores the existence of regional variations in treatment strategies in PACG patients, highlighting the need for broader promotion and standardisation of guidelines to unify clinical decision-making.

Our results show that patients with higher IOP and worse visual acuity had lower LPI usage rates. A prior study found that elevated baseline IOP before LPI correlates with an increased risk of postoperative persistent IOP elevation and progressive optic neuropathy in PACG patients [34]. Worse visual acuity often indicates more severe optic nerve damage, which may limit the chances of benefitting from LPI. For these advanced cases, the low LPI rate may reflect a conscious clinical decision: ophthalmologists may consider isolated LPI insufficient for severe disease and thus intend to perform surgery, yet such intervention is often deferred to subsequent hospitalisations due to medical insurance restrictions, which only allow reimbursement for one eye per surgical admission. Notably, these patients were discharged without receiving definitive intervention, which is concerning given the low follow-up adherence among glaucoma patients in China [35]. Ophthalmologists should proactively explain its necessity to patients to avoid missed opportunities for intervention. IOP and visual acuity were associated with LPI use, but these associations likely reflect confounding by disease severity rather than independent causal determinants. Our retrospective design cannot fully explain the underlying reasons for low utilization. Future prospective studies or clinical audits that systematically capture gonioscopic findings and document surgeon reasoning are needed to truly understand the barriers to LPI implementation and guide targeted interventions.

The findings of this study must be interpreted within the specific context of China’s healthcare system, where hospitalisation is typically required for definitive PACG treatment and medical insurance reimbursement is generally limited to one eye per surgical admission [36]. These systemic features fundamentally influence clinical decision-making. Consequently, the 35.3% LPI uusage rate observed here reflects not only clinical judgment but also administrative and financial constraints specific to China. However, China bears the world’s largest PACG patient population [1] and a high blindness rate [15]. Understanding real-world treatment patterns within its unique healthcare system carries irreplaceable global public health significance. The ‘treatment gap’ identified in this study, namely significant underutilization persisting even within a structurally favorable inpatient setting, suggests that barriers to guideline-concordant care may be partially independent of healthcare delivery models, stemming from more universal factors such as clinical cognition, resource allocation, or patient-level barriers. These findings provide important reference for other high-burden regions, particularly in Asia, and offer empirical evidence for global health policymakers on the disparity between ‘structural accessibility’ and ‘actual utilization’.

This study has several strengths. First, the nationally representative sample, which was recruited from various hospitals across different regions, ensured generalisability to China’s hospitalised PACG patients. Second, it is the first study to systematically report LPI use in unoperated eyes, providing valuable evidence for informing PACG management policies. However, this study also has several limitations. First, we did not take further steps to determine the reasons why patients did not receive LPI. Second, a notable proportion of patients may have received LPI after discharge due to medical insurance restrictions or delayed clinical decisions, so we may have underestimated the real usage rates of LPI. Third, the diagnostic heterogeneity of glaucoma may exist because of the differing diagnostic criteria of different levels of hospitals. Finally, this retrospective study is limited by a severe lack of gonioscopy data. This finding based on the PAS results should be interpreted with caution given the limited sample size and requires confirmation in prospective studies. This also indirectly reflects the underutilisation of gonioscopy in the clinical management of PACG patients.

## Conclusion

This national multi-centre study investigated the usage rate and influencing factors of LPI among hospitalised PACG patients with unoperated eyes. We found a low LPI usage rate in the unoperated eyes of hospitalised PACG patients. Hospital region, visual acuity and IOP were factors that influenced LPI use. Therefore, it is critical to enhance guideline-based training for ophthalmologists and improve patients’ health literacy, which will help align clinical practice with guideline recommendations and reduce visual impairment and blindness in PACG patients.

## Electronic Supplementary Material

Below is the link to the electronic supplementary material.

Supplementary Material 1

## Data Availability

Data are available upon reasonable request and contact the corresponding author.

## Abbreviations

PACG: Primary angle-closure glaucoma
LPI: Laser peripheral iridotomy
EMR: Electronic medical record
LPIP: Laser peripheral iridoplasty
PAS: Peripheral anterior synechiae
IOP: Intraocular pressure
GEE: Generalized estimating equations
