# Supplementary material for: Laser peripheral iridectomy use in the unoperated eyes of hospitalised patients with primary angle-closure glaucoma in a national multicentre study in China
Source: BMC Ophthalmol. 2026 May 7;26:365. doi: 10.1186/s12886-026-04873-y (PMC13321662; doi:10.1186/s12886-026-04873-y)
Supplement: Supplementary file 1 — Supplementary Material 1 [file 12886_2026_4873_MOESM1_ESM.docx]

**Supplementary Materials**

**Table S1.** Participating hospitals and the number of included patients with PACG.

| **Region** | | **Participating hospital** | **Number of cases** | **Participating hospital** | **Number of cases** |
| --- | --- | --- | --- | --- | --- |
|  |  | **Provincial level or above hospitals (N = 14)** | **3,900 cases** | **City-level hospital (N = 12)** | **1,504 cases** |
| Nationally-leading ophthalmic hospital | - | Tianjin Medical University Eye Hospital | 295 | - | - |
|  |  | The Eye Hospital, Wenzhou Medical University | 291 | - | - |
| North East | Jilin | Jilin University Second Hospital | 269 | Jilin Central General Hospital | 148 |
|  |  | Jilin Provincial People’s Hospital | 299 | Liaoyuan City Central Hospital | 150 |
| North | Hebei | The Second Hospital of Hebei Medical University | 272 | Handan City Eye Hospital | 150 |
|  |  | Hebei General Hospital | 294 | Hebei Eye Hospital | 148 |
| Eastern | Jiangsu | The First Affiliated Hospital of Soochow University | 300 | Lixiang Eye Hospital of Soochow University | 144 |
|  |  | Jiangsu Province Hospital | 298 | Huaiyin Hospital of huai’an city | 149 |
| Central-South | Hunan | Xiangya Hospital of Central South University | 292 | Yueyang Central Hospital | 141 |
|  |  | Hunan Provincial People’s Hospital | 296 | Zhangjiajie People’s Hospital | 145 |
| South West | Sichuan | West China Hospital of Sichuan University | 283 | Ziyang Hospital of Traditional Chinese Medicine | 92 |
|  |  | Sichuan Provincial People’s Hospital | 290 | Mianyang Central Hospital | 149 |
| North West | Gansu | Lanzhou University Second Hospital | 290 | Baiyin City Central Hospital | 58 |
|  |  | Gansu Provincial Hospital | 131 | Zhangye People’s Hospital | 30 |

The number of cases in all hospitals was determined after excluding those not eligible for the PACG diagnosis or patients aged < 18 years. A limited number of PACG in-patients were in some hospitals from 2011 to 2020: Gansu Provincial Hospital only has 131 cases of patients with PACG; Ziyang Hospital of Traditional Chinese Medicine only has 92 cases of patients with PACG; Baiyin City Central Hospital only has 58 cases of patients with PACG; Zhangye People's Hospital only has 30 cases of patients with PACG.
